# Single-cell analysis reveals potential therapeutic markers of peripheral blood mononuclear cells from bladder cancer patients

**DOI:** 10.1590/1414-431X2025e14002

**Published:** 2025-05-09

**Authors:** Xingning Mao, Rirong Yang, Yunkun Yan, Yanyu Zeng, Mengying Bao, Rong Huang, Yan Dai, Qingyun Zhang, Yu Ye, Jiwen Cheng, Zengnan Mo, Haiying Zhang

**Affiliations:** 1 Center for Genomic and Personalized Medicine, Guangxi Key Laboratory for Genomic and Personalized Medicine, Guangxi Collaborative Innovation Center for Genomic and Personalized Medicine, Guangxi Medical University, Nanning, Guangxi, China; 2 Institute of Urology and Nephrology, First Affiliated Hospital of Guangxi Medical University, Guangxi Medical University, Nanning, Guangxi, China; 3 Department of Immunology, School of Basic Medical Sciences, Guangxi Medical University, Nanning, Guangxi, China; 4 Department of Urology, the First Affiliated Hospital of Guangxi Medical University, Guangxi Medical University, Nanning, Guangxi, China; 5 Collaborative Innovation Centre of Regenerative Medicine and Medical BioResource Development and Application Co-constructed by the Province and Ministry, Guangxi Medical University, Nanning, Guangxi, China; 6 Emergency Department, the Second Affiliated Hospital of Guangxi Medical University, Guangxi Medical University, Nanning, Guangxi, China; 7 Department of Urology, Affiliated Tumor Hospital of Guangxi Medical University, Guangxi Medical University, Nanning, Guangxi, China; 8 Department of Occupational Health and Environmental Health, School of Public Health, Guangxi Medical University, Nanning, Guangxi, China

**Keywords:** Bladder cancer, Peripheral blood mononuclear cells, Immunotherapy targets, Single-cell RNA sequencing analysis, Immune subtype

## Abstract

Bladder cancer is the most prevalent malignancy of the urinary tract, with significant advancements in treatment achieved over recent decades. Nonetheless, the immunological mechanisms underlying bladder cancer progression remain elusive, and only a limited number of patients derive benefit from current immune checkpoint inhibitors. Here, we conducted a single-cell RNA sequencing analysis of 44,022 cells from peripheral blood mononuclear cell samples of bladder cancer patients and a healthy donor. Our findings indicated that the proportions of T cells and neutrophils are higher in bladder cancer patients than in the healthy donor. *LAG3*, *HAVCR2*, and *CTLA4* had elevated expression levels in CD8-T2-GZMK cell clusters from patients. CD8-T7-STMN1 cells highly expressed *ITGAE*, *CD38*, and *STMN*1. Furthermore, NK3-CMC1, more prevalent in patients, showed a high expression of *TIGIT.* Additionally, Bcell2-TCL1A and Bcell3-MS4A1 were characterized by the high expression of inhibitory receptor marker genes. Gene set variation analysis suggested that Mono4-THBS1 may play a role in promoting tumor hypoxia and angiogenesis. Neu-FCGR3B exhibited high levels of *IL4R* and *CD274* expression. Our study indicated that LAG-3 and TIM-3 may serve as novel potential immune checkpoint inhibitors in bladder cancer treatment. The phenotypes of NK3-CMC1, Bcell2-TCL1A, and Bcell3-MS4A1 might be altered by tumor progression. Mono4-THBS1 could potentially be a source of tumor-enriched monocyte-like cells. Neu-FCGR3B may play a detrimental role in the anti-tumor response and could emerge as a predictive marker for bladder cancer. Overall, these high-resolution transcriptomic data offer invaluable insights for identifying new therapeutic targets and biomarkers in bladder cancer immunotherapy.

## Introduction

Bladder cancer, the most common malignancy of the urinary tract, affected over 570,000 individuals globally in 2020 (1,2). Urothelial cancer, representing the predominant histological subtype of bladder malignancies, typically presents in two forms: non-muscle-invasive bladder cancer (NMIBC) or muscle-invasive bladder cancer (MIBC) (3). NMIBC accounts for 70% of new bladder cancer cases, characterized by tumors confined to the urothelium or lamina propria. Notably, around 10 to 20% of NMIBCs progress to MIBC, which is associated with a poor prognosis (1). Over recent decades, advancements in understanding bladder cancer biology have led to significant progress in its treatment, including the use of immune checkpoint inhibitors (ICIs) (2,4). Despite these advancements, the prognosis for patients with metastatic urothelial cancer remains poor, with only a limited number of patients benefiting from current ICIs (3,5). This underscores the urgent need to develop novel, targeted, and more effective therapies for bladder cancer.

Cancer has been established as a systemic disease, as evidenced by its induction of systemic responses, including the regulation of immune function (6). Alterations in immune cells have been observed in the peripheral blood of cancer patients (7). The recirculation of tumor-resident immune cells, or alterations in peripheral immune cells detected in cancer patients' blood, may yield insights into cancer pathology (8). Given the ease of obtaining peripheral blood samples compared to tissue samples, profiling peripheral blood in cancer patients could hold clinical significance (8). The recent advent of single-cell RNA sequencing (scRNA-seq) has offered powerful tools for dissecting the heterogeneity of immune cells in various cancers (9). Furthermore, scRNA-seq has enabled the identification of potential cellular targets for immunotherapy and rare immune cell populations in the peripheral blood of patients (10,11). Nevertheless, the application of scRNA-seq in researching the peripheral blood of bladder cancer patients remains limited.

In this study, we delineated the landscape of immune cells and identified characteristics of specific immune cell subsets by performing scRNA-seq on peripheral blood mononuclear cells (PBMCs) from bladder cancer patients and a healthy donor. Our high-resolution transcriptome data could be instrumental in developing new targets for immunotherapy in bladder cancer.

## Material and Methods

### Enrollment of participants and sample collection

Our study adhered to the principles of the Declaration of Helsinki (as revised in 2013). Peripheral blood samples were collected from four patients prior to surgery and from a healthy donor during a routine check-up. All patients were treatment-naive (Supplementary Table S1). PBMCs were isolated using lymphocyte separation medium (Human) (Solarbio, China). Cell viability was assessed using trypan blue staining, and only samples with a cell viability rate above 80% were used for single-cell library construction.

### Library preparation and sequencing

The single-cell suspension was processed into uniquely barcoded RNA using Chromium Single-Cell 3′ Reagent Kits (v3 chemistry,10X Genomics, USA), in accordance with the manufacturer's instructions. The single-cell library underwent sequencing on Illumina NovaSeq 6000 instruments (Illumina, USA), employing 150-bp paired-end sequencing.

### Raw data preprocessing and quality control

FASTQ data obtained from the sequencing output were aligned and quantified against the human reference genome (GRCh38) using Cell Ranger software (pipeline version 3.1.0, 10X Genomics), employing default parameters. For validation, we incorporated additional PBMC scRNA-seq data from other two healthy donors obtained from the 10× Genomics platform into our original dataset. Subsequently, the feature-barcode matrix was converted into a Seurat object utilizing the R package Seurat (https://satijalab.org/seurat/). Cells of low quality were excluded based on the following criteria: feature counts exceeding 5000 or less than 300 and mitochondrial gene percentage greater than 15%. After filtering, the feature-barcode matrix for each sample was normalized using the “NormalizeData” function with default settings (“LogNormalize” method and scale.factor=10,000), followed by the identification of the top 2,000 highly variable genes (HVFs) employing the “FindVariableFeatures” function (“vst” method). Subsequently, doublets in each sample were identified utilizing the R package DoubletFinder, assuming a 5% doublet formation rate.

### Dimensionality reduction and clustering

After quality control, the datasets from all samples were integrated using Seurat to mitigate batch effects. The integrated dataset underwent normalization, followed by the identification of HVFs using Seurat. Variables such as “percent.mito” and “nCount_RNA” were regressed out employing the “ScaleData” function. Subsequently, principal component analysis (PCA) was conducted using the “RunPCA” function. The top 50 principal components were utilized for dimensionality reduction to visualize cells using the “RunTSNE” function. Major clusters were identified through the “FindNeighbors” and “FindClusters” functions, set at a resolution of 1.4. Differentially expressed genes within each cluster were determined using the “FindAllMarkers” function. The dataset was categorized into T cells, natural killer (NK) cells, B cells, monocytes, dendritic cells (DCs), and neutrophils based on established markers: *CD3D*, *CD3E*, *CD3G* for T cells; *NKG7*, *GNLY*, *KLRB1* for NK cells; *CD79A*, *CD79B*, *MS4A1* for B cells; *CD68*, *CD14*, *MS4A7* for monocytes; *CST3*, *FCER1A*, *CD1C* for DCs; *S100A8*, *S100A9*, *FCGR3B* for neutrophils. Subsequently, T cells, NK cells, and B cells underwent further re-clustering. Cells expressing dual-lineage genes were excluded from downstream analysis. The re-clustering process encompassed normalization, HVF identification, data scaling, PCA, batch effect elimination using the “harmony” package, t-distributed stochastic neighbor embedding (tSNE) visualization, and cluster identification.

### Gene ontology (GO) analysis

The top 100 to 150 genes from each cluster were selected for GO analysis. This analysis was conducted using the “enrichGO” function of the “clusterProfiler package”, with default parameters applied.

### RNA velocity analysis

RNA velocity analysis was conducted using the velocyto.R program. Initially, velocyto.py was used to annotate spliced/unspliced reads for each sample using the possorted_genome_bam.bam file, produced by Cell Ranger (USA), and the results were saved in a loom file. Subsequently, the loom files for each sample were imported into R and merged to create count tables containing both spliced and unspliced reads. Cells representing the lowest 0.5% in total unspliced transcript count were then excluded. Furthermore, genes exhibiting an average spliced variant expression below 0.2 or an average unspliced variant expression below 0.05 in at least one cluster were eliminated. Lastly, arrows displaying RNA velocity information were incorporated into the tSNE plot generated via Seurat.

### Identification of gene regulatory network and specific transcription factors

The identification of the gene regulatory network and specific transcription factors was accomplished through the utilization of the SCENIC package. Coexpression modules were deduced utilizing the “runGENIE3” function, and potential direct binding targets of transcription factors, known as regulons, were discerned using the human motif database within a 10 kb radius of the transcription start site (TSS) for RcisTarget. Subsequently, the activity of these regulons in cell clusters was assessed using AUCell and then averaged.

### Differentially expressed gene analysis between patients and healthy donor

Differentially expressed genes between patients and the healthy donor were identified using the Seurat “FindMarkers” function. A gene was deemed significant if its adjusted P value was less than 0.05. The volcano plot illustrating these genes was created using the ggplot2 package.

### Gene set variation analysis (GSVA)

Gene sets representing pathways were obtained from the GSEA database. Pathway scores were computed using the GSVA package and visualized with the ggplot2 package.

## Results

### Landscape of PBMCs from the patients with bladder cancer and healthy donor profiled by scRNA-seq analysis

**Figure 1:**
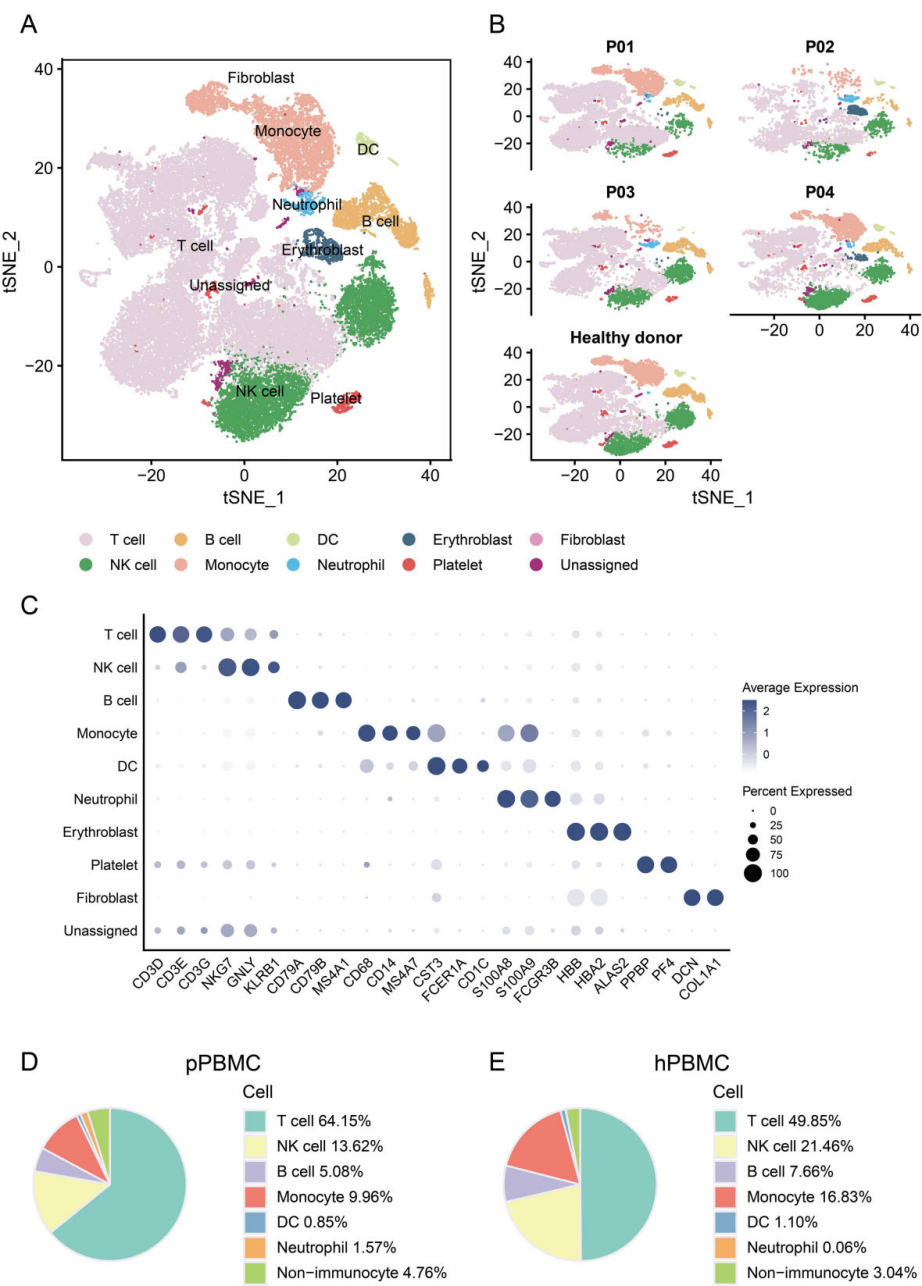
Landscape of peripheral blood mononuclear cells (PBMCs) from bladder cancer patients and the healthy donor profiled by scRNA-seq analysis. **A**, tSNE plot depicting single cells, categorized by dominant cell types. **B**, tSNE plot representing all cells, differentiated by sample origin. **C**, Dot plot illustrating the expression levels of defining markers, with color denoting average expression. **D** and **E**, Pie plots displaying the proportions of major cell types originating from PBMCs of patients (**D**) and the healthy donor (**E**). pPBMC: PBMCs of patients; hPBMC: PBMCs of healthy donor; DC: dendritic cells.

We analyzed four PBMC samples from bladder cancer patients and one from a healthy donor to map the PBMC landscape. Each sample underwent individual scRNA-seq processing. Following rigorous quality control and filtration, a total of 44,022 cells were collected, comprising 33,816 cells from patients and 10,206 cells from the healthy donor. These scRNA-seq data were then integrated into a single dataset for unsupervised graph-based clustering (Figure 1A). Consistent immune cell types were observed across all samples (Figure 1B). We identified various immune cells, including T cells, NK cells, B cells, monocytes, DCs, and neutrophils, using canonical cell markers (Figure 1C). Notably, increased proportions of T cells and neutrophils were observed in patients compared to the healthy donors (Figure 1D and E, Supplementary Figure S1). Our study provided a comprehensive atlas of PBMCs, highlighting differential immune cell type distributions between bladder cancer patients and healthy donors.

### Heterogeneity and dynamics of T cell subpopulations revealed by scRNA-seq analysis

**Figure 2:**
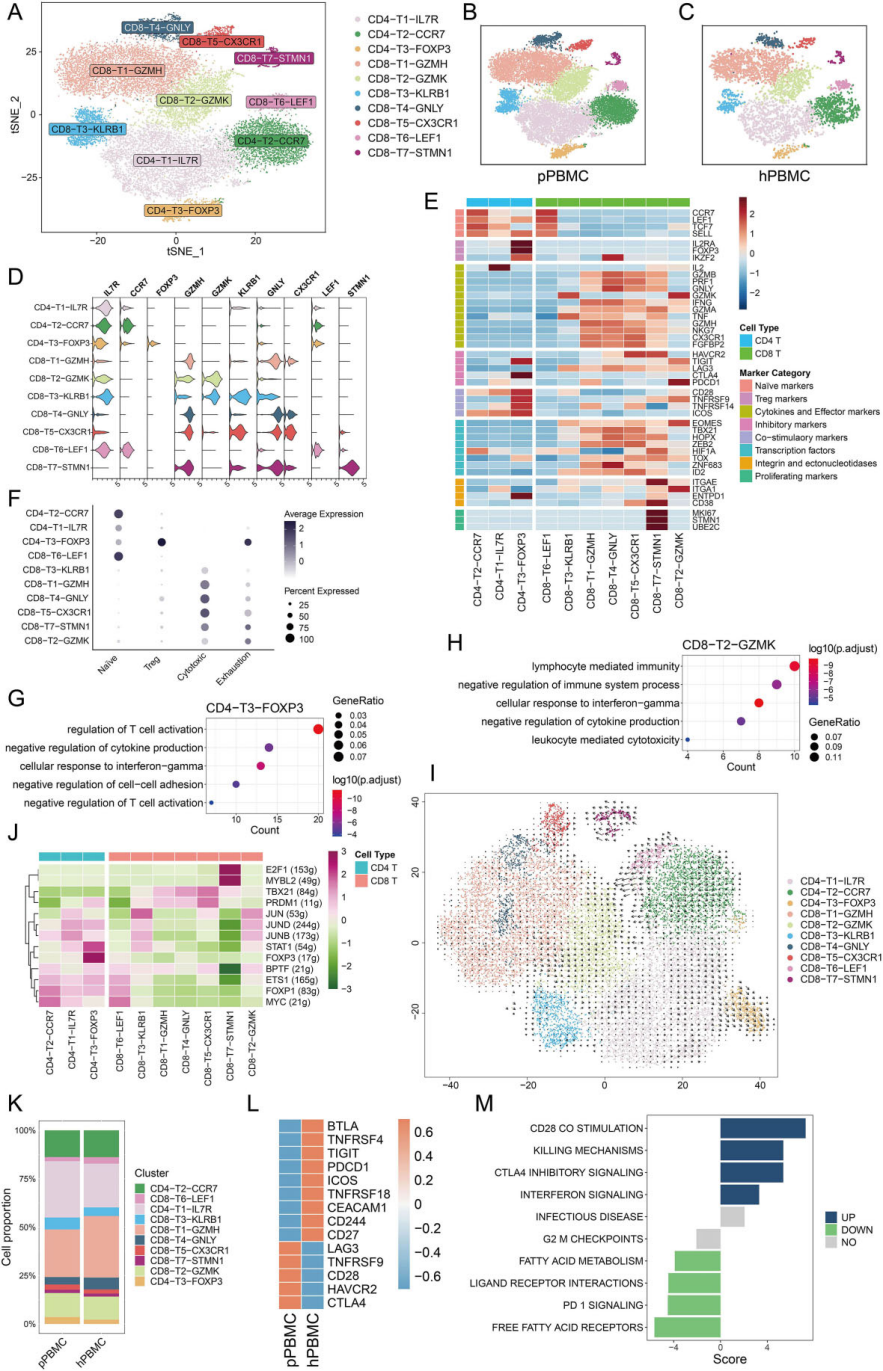
Heterogeneity and dynamics of T cell subtypes revealed by scRNA-seq analysis. **A**, tSNE plot showing T cell clusters. **B** and **C**, tSNE plot delineating T cell clusters derived from peripheral blood mononuclear cells (PBMCs) of patients (pPBMC) (**B**) and the healthy donor (hPBMC) (**C**). **D**, Violin plot depicting the expression profiles of T cell cluster markers. **E**, Heatmap illustrating the expression levels of various marker genes across T cell clusters, including naive T cell markers, regulatory T cell markers, cytokines, effective, inhibitory, co-stimulatory marker genes, transcription factors, integrins, ectonucleotidases, and proliferating markers. Red: upregulation; blue: downregulation. **F**, Dot plot of representative cytotoxic, exhaustive, and naive signatures in T cell clusters, with Z-score normalized log2 (count+1) data. **G** and **H**, GO analysis for CD4-T3-FOXP3 (**G**) and CD8-T2-GZMK (**H**). **I**, Visualization of the transition among T cell clusters based on RNA velocity analysis. **J**, Regulon analysis of T cell clusters, displaying transcription factors (rows) and their expression in different T cell clusters (columns). Purple: upregulation; green: downregulation. **K**, Proportions of T cell clusters in original samples from patients and the healthy donor. **L**, Heatmap showing the comparative expression levels of immune target genes in CD8-T2-GZMK between pPBMC and hPBMC samples. Orange: upregulation; blue: downregulation. **M**, Heatmap displaying the GSVA scores for canonical pathway gene sets enriched in CD8-T2-GZMK from patients and the healthy donor. Blue: upregulation; green: downregulation.

Given the predominance of T cells in PBMCs, we initially focused on dissecting T cell subpopulations through re-clustering analysis (Figure 2A-C). We identified three CD4 T cell clusters and seven CD8 T cell clusters (Figure 2D). CD4-T1-IL7R, characterized by high *IL2* expression, was annotated as CD4 helper T cells (Figure 2E). CD4-T2-CCR7 exhibited elevated expression of naive T cell signature genes, including *CCR7*, *TCF7*, *LEF1*, and *SELL* (Figure 2E). CD4-T3-FOXP3 showed pronounced expression of regulatory T cell (Treg) signature genes (*IL2RA*, *FOXP3*, *IKZF2*) and co-stimulatory markers (*CD28*, *TNFRSF9*, *TNFRSF14*, *ICOS*) (Figure 2E). In six CD8 T cell clusters, cytokines and effector genes were notably expressed (CD8-T1-GZMH, CD8-T2-GZMK, CD8-T3-KLRB1, CD8-T4-GNLY, CD8-T5-CX3CR1, CD8-T7-STMN1) (Figure 2E). Three of these clusters (CD8-T2-GZMK, CD8-T5-CX3CR1, CD8-T7-STMN1) additionally showed variable expression of inhibitory markers. CD8-T2-GZMK predominantly expressed *PDCD1* and *TIGIT*, whereas CD8-T5-CX3CR1 and CD8-T7-STMN1 expressed *HAVCR2* (Figure 2E). Notably, CD8-T7-STMN1 exhibited high levels of *ITGAE* (*CD103*) and *CD38*, indicative of tissue-resident T cell phenotypes (Figure 2E). Similar to CD4-T2-CCR7, CD8-T6-LEF1 exhibited high expression of *CCR7*, *LEF1*, *TCF7*, and *SELL* (Figure 2E). Collectively, our findings revealed the heterogeneity of T cell subpopulations within PBMCs.

To confirm the phenotypes of various T cell subpopulations, we utilized publicly available signatures for naive, Treg, cytotoxic, and exhausted T cells, applying these to all T cell clusters to calculate a transcriptional score. CD4-T2-CCR7 and CD8-T6-LEF1 exhibited the highest naive scores, indicative of naive T cell phenotypes. CD4-T3-FOXP3 displayed the most pronounced Treg signatures, and is thus classified as Tregs. Notably, CD8-T2-GZMK and CD8-T7-STMN1 demonstrated the most significant exhaustion scores, correlating with exhausted T cell (Texs) phenotypes (Figure 2F).

We subsequently investigated the roles of Tregs and Texs within PBMCs by conducting GO analysis on CD4-T3-FOXP3 and CD8-T2-GZMK. Both clusters demonstrated enrichment in “negative regulation of cytokine production” and “cellular response to interferon-gamma” (Figure 2G and H). Additionally, CD8-T2-GZMK showed enrichment in “lymphocyte mediated immunity” and “negative regulation of immune system process” (Figure 2H).

Zheng et al. (12) identified a transition in T cell states from activation to exhaustion in hepatocellular carcinoma. To further explore this transition in T cell clusters of PBMCs, we utilized RNA velocity analysis. This analysis revealed a directional flow from CD4-T2-CCR7 to CD4-T1-IL7R, subsequently leading to CD4-T3-FOXP3 (Figure 2I). The findings suggested that CD4-T1-IL7R represents an intermediate state between naive T cells and Tregs. In the context of CD8 T cells, both CD8-T6-LEF1 and CD8-T1-GZMH demonstrated a lineage trajectory towards CD8-T2-GZMK (Figure 2I). These observations elucidated the dynamic states of various T cell subpopulations.

Next, we endeavored to pinpoint the transcription factors associated with various states of T cell subpopulations through SCENIC analysis. Notably, JUN, JUND, and JUNB exhibited increased activity in CD8-T2-GZMK, whereas E2F1 and MYBL2 demonstrated elevated activities in CD8-T7-STMN1 (Figure 2J). These findings further highlight the heterogeneity within the Texs of PBMCs.

Finally, we conducted a comparative analysis of the T cell subpopulations between patients and the healthy donor. CD4-T1-IL7R, CD4-T3-FOXP3, and CD8-T3-KLRB1 were more prevalent in patients (Figure 2K). Given that CD8-T2-GZMK was characterized as Texs, we proceeded to compare the expression levels of immune target genes in CD8-T2-GZMK between patients and the healthy donor. Notably, *LAG3*, *HAVCR2*, and *CTLA4* exhibited higher expression levels in CD8-T2-GZMK from patients (Figure 2L). Utilizing GSVA, we discerned that CD8-T2-GZMK from patients preferentially expressed genes associated with CTLA-4 inhibitory signaling and interferon signaling (Figure 2M). Taken together, these findings suggested potential immunotherapeutic targets for bladder cancer.

### Dissection and clustering of NK cells

**Figure 3:**
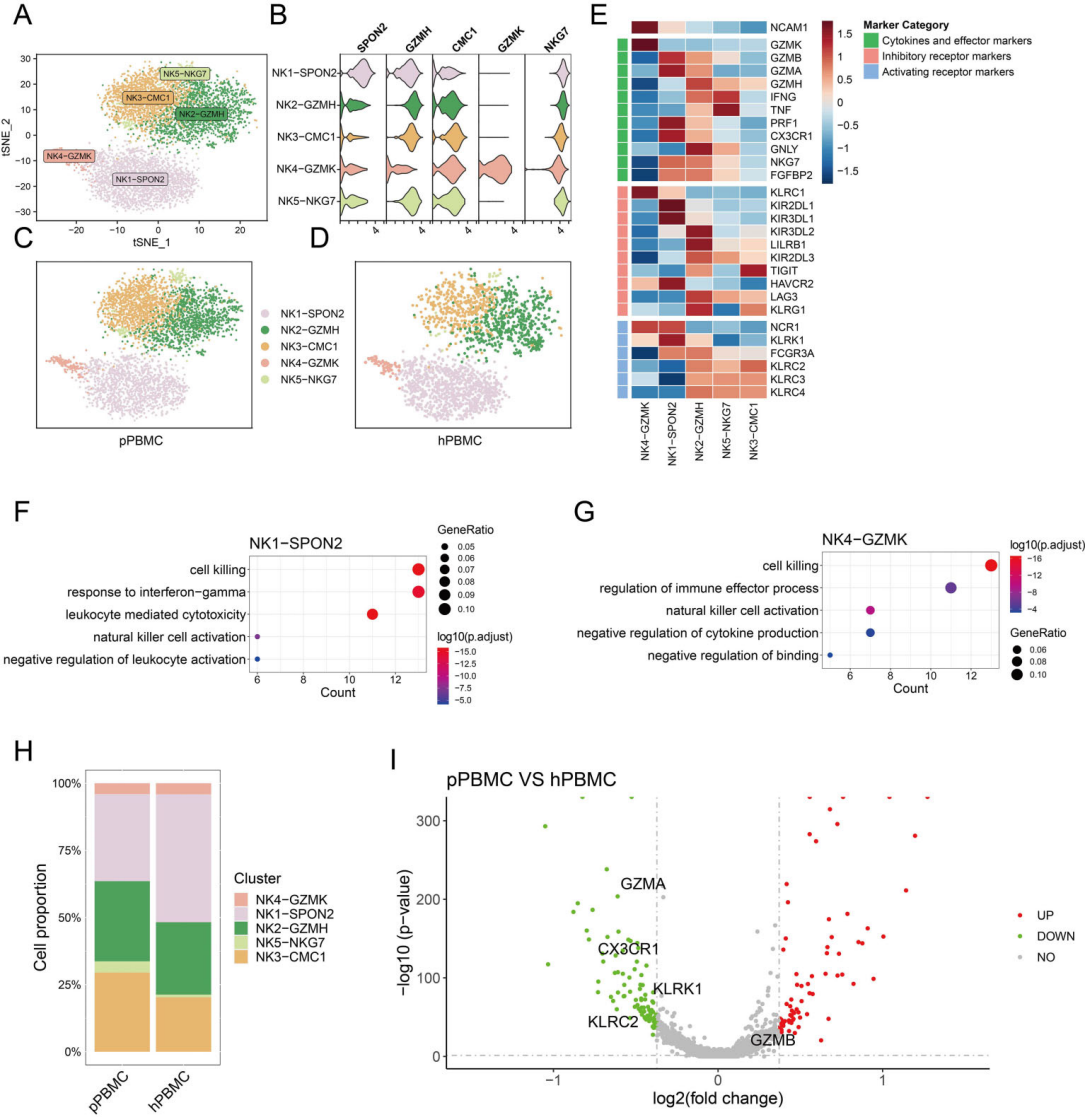
Dissection and clustering of natural killer (NK) cells. **A**, tSNE plot displaying NK cell clusters. **B**, Violin plot illustrating the expression profiles of NK cell cluster markers. **C** and **D**, tSNE plot highlighting NK cell clusters derived from peripheral blood mononuclear cells (PBMCs) of patients (pPBMC) (**C**) and the healthy donor (hPBMC) (**D**). **E**, Heatmap depicting the expression levels of cytokines and effective marker genes, alongside inhibitory and activating receptor marker genes among NK cell clusters. Red: upregulation; blue: downregulation. **F** and **G**, GO analysis for NK1-SPON2 (**F**) and NK4-GZMK (**G**). **H**, Proportions of NK cell clusters of pPBMC and hPBMC samples. **I**, Volcano plot showing the expression levels of differentially expressed genes in NK cells from patients and the healthy donor. Red: upregulation; green: downregulation.

To investigate the characteristics of NK cells within PBMCs, we conducted unsupervised re-clustering analysis (Figure 3A-D). Within the five identified clusters, NK4-GZMK was distinguished by the highest expression level of *NCAM1* (CD56) and low expression levels of cytokines and effect marker genes, indicative of CD56^bright^ NK cell phenotypes (Figure 3E). Three clusters (NK2-GZMH, NK5-NKG7, NK3-CMC1) exhibited high expression of cytokines and activating receptor marker genes, which were annotated as mature NK cells (Figure 3E). NK1-SPON2 displayed a moderate expression level of *NCAM1*, coupled with the expression of both inhibitory and activating receptor marker genes (Figure 3E), suggesting that NK1-SPON2 may represent an intermediate state between CD56^bright^ NK cells and mature NK cells. Subsequent GO analysis was utilized to delve deeper into the functions of these NK cells. Both NK1-SPON2 and NK4-GZMK were enriched in genes associated with “cell killing, natural killer cell activation” (Figure 3F and G). Specifically, NK1-SPON2 harbored genes implicated in the “negative regulation of leukocyte activation”, whereas NK4-GZMK was enriched in genes linked to the “negative regulation of cytokine production, negative regulation of binding” (Figure 3F and G).

Next, we sought to delineate the disparities in NK cell subpopulations between patients and the healthy donor. Notably, the cell proportions of NK3-CMC1 and NK5-NKG7 were elevated in patients, whereas NK1-SPON2 showed an increased prevalence in the healthy donor (Figure 3H). In terms of gene expression, *GZMB* was more highly expressed in NK cells from patients, while *CX3CR1*, *GZMA*, *KLRK1*, and *KLRC2* demonstrated higher expression levels in NK cells from the healthy donor (Figure 3I). Altogether, these findings contributed to an atlas of NK cell subpopulations within PBMCs.

### Deciphering diversity of B cell subpopulations

**Figure 4:**
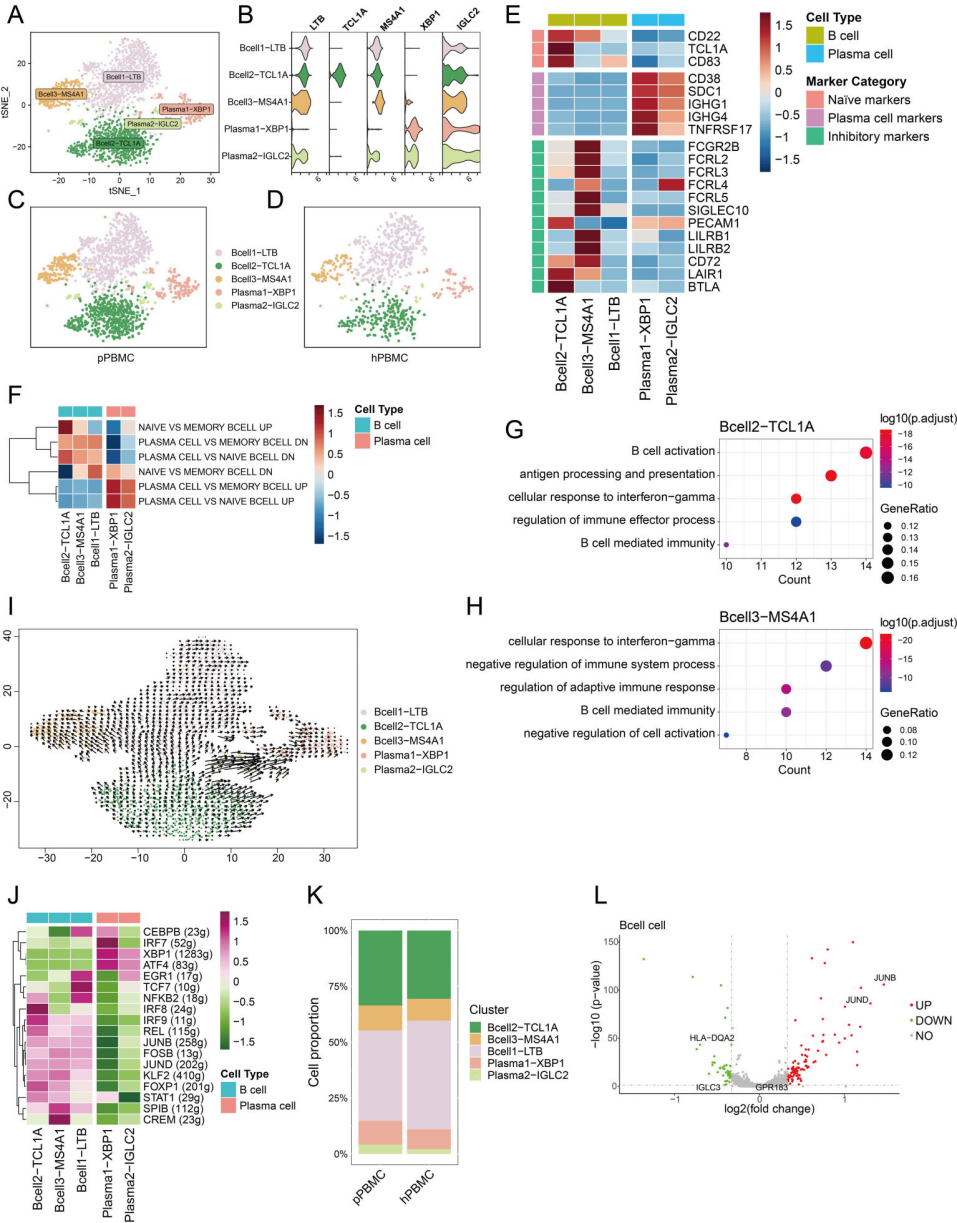
Deciphering diversity of B cell subpopulations. **A**, tSNE plot illustrating B cell clusters. **B**, Violin plot depicting the expression profiles of B cell cluster markers. **C** and **D**, tSNE plot delineating B cell clusters derived from peripheral blood mononuclear cells of patients (pPBMCs) (**C**) and the healthy donor (hPBMCs) (**D**). **E**, Heatmap depicting the expression levels of naive B cell markers, plasma cell markers, and inhibitory marker genes among B cell clusters. Red, upregulation; blue, downregulation. **F**, Heatmap showing the gene set variation analysis (GSVA) scores for gene sets related to B cell pathways enriched in B cell clusters. Red, upregulation; blue, downregulation. **G** and **H**, Gene ontology (GO) analysis for Bcell2-TCL1A (**G**) and Bcell3-MS4A1 (**H**). **I**, Visualization of B cell cluster transitions based on RNA velocity analysis. **J**, Regulon analysis of B cell clusters, with rows representing transcription factors and columns indicating B cell clusters. Purple: upregulation; green: downregulation. **K**, Proportions of B cell clusters in pPBMC and hPBMC. **L**, Volcano plot demonstrating the expression levels of differentially expressed genes in B cells from patients and the healthy donor. Red: upregulation; green: downregulation.

Following the re-clustering of B cells from PBMCs, we identified a total of 2358 cells across five distinct clusters (Figure 4A-D). Bcell2-TCL1A was notable for its high expression of *CD22*, *TCL1A*, and *CD83*, indicative of a naive B cell phenotype (Figure 4E). Intriguingly, the majority of the inhibitory receptor marker genes were found to be preferentially expressed in Bcell3-MS4A1 (Figure 4E). Additionally, two clusters, Plasma1-XBP1 and Plasma2-IGLC2, exhibited high expression levels of plasma cell marker genes, annotated as plasma B cells (Figure 4E).

To further gain insights into the function of B cell subpopulations, we performed GSVA and GO analysis. Bcell2-TCL1A was found to encompass genes associated with “naive *vs* memory B cell up, cellular response to interferon-gamma, regulation of immune effector process”, showing that Bcell2-TCL1A could be responsive to interferon-gamma and play a role of immune regulation (Figure 4F and G). Moreover, Bcell3-MS4A1 was enriched in genes related to “naive *vs* memory B cell down, cellular response to interferon-gamma, negative regulation of immune system process and cell activation”, suggesting that Bcell3-MS4A1 may also respond to interferon-gamma (IFN-γ) and have an inhibitory role in immunity (Figure 4F and H).

Subsequently, we employed velocity analysis to elucidate the developmental trajectory of B cells. This analysis revealed a directional progression from Bcell2-TCL1A to Bcell1-LTB, and subsequently to Bcell3-MS4A1 (Figure 4I). This pattern suggested that Bcell1-LTB may represent an intermediate state between naive B cells and memory B cells. To further investigate the key transcription factors (TFs) involved in this transition, we conducted SCENIC analysis. The results showed elevated activities of CEBPB, EGR1, TCF7, and NFKB2 in Bcell1-LTB, whereas SPIB and CREM activity significantly increased in Bcell3-MS4A1 (Figure 4J).

Finally, we evaluated the differences in B cell subpopulations between patients and the healthy donor. The cell proportions of Bcell2-TCL1A and Bcell3-MS4A1 were found to be increased in patients, whereas an increase in Bcell1-LTB was observed in the healthy donor (Figure 4K). In terms of gene expression, *JUNB* and *JUND* were upregulated in B cells derived from patients, while *HLA-DQA2* and *IGLC3* exhibited higher expression levels in B cells from the healthy donor (Figure 4L). Collectively, these findings provide a comprehensive overview of the diverse characteristics of B cell subpopulations within PBMCs.

### Distinct functional composition of myeloid cells in PBMCs

**Figure 5:**
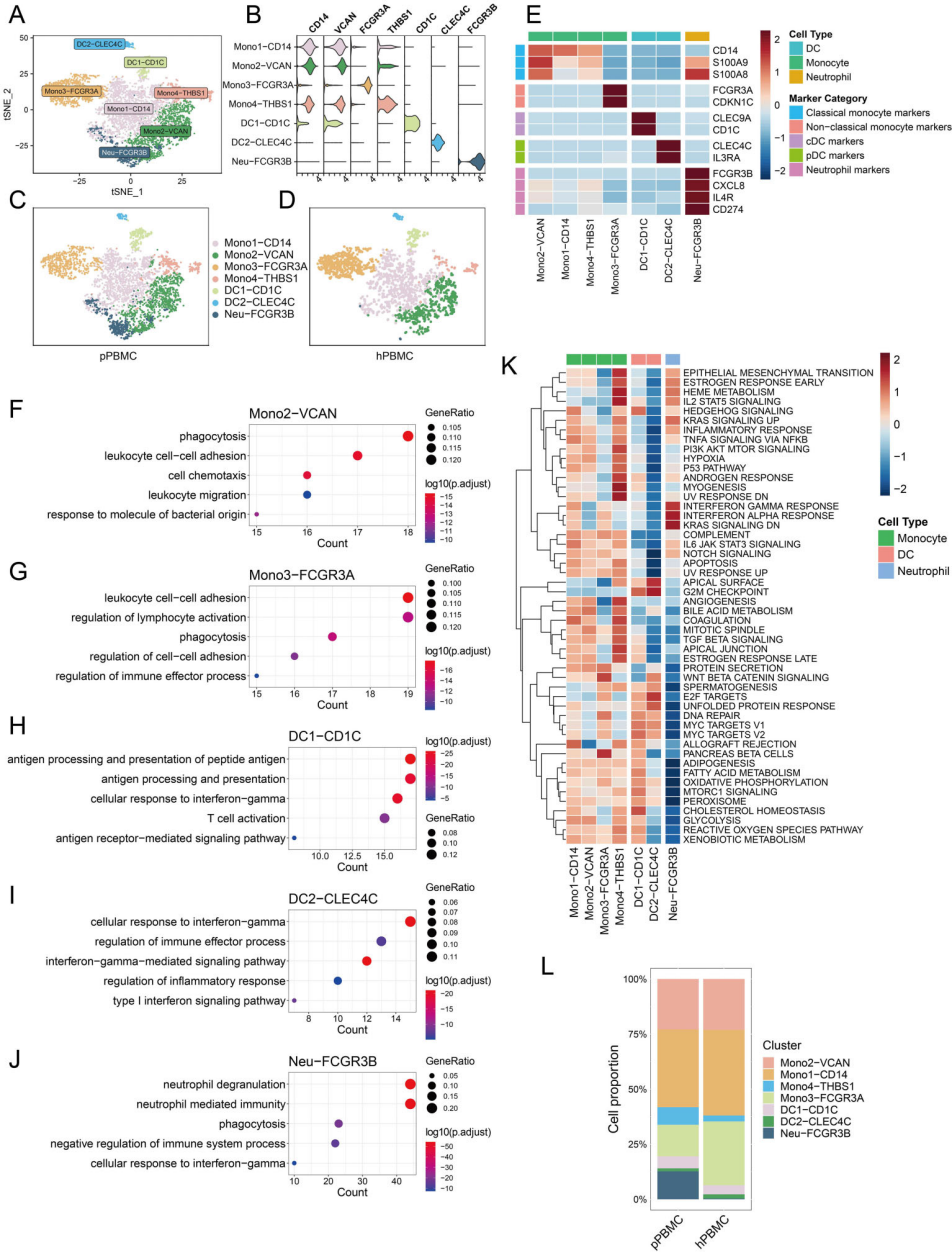
Distinct functional composition of myeloid cells in peripheral blood mononuclear cells (PBMCs). **A**, tSNE plot depicting myeloid cell clusters. **B**, Violin plot illustrating the expression profiles of markers specific to myeloid cell clusters. **C** and **D**, tSNE plot highlighting myeloid cell clusters derived from PBMCs of patients (pPBMC) (**C**) and the healthy donor (hPBMC) (**D**). **E**, Heatmap displaying the expression levels of marker genes across myeloid cell clusters, including classical monocyte, non-classical monocyte, cDC, pDC, and neutrophil marker genes. Red: upregulation; blue: downregulation. **F**-**J**, Gene ontology (GO) analysis for Mono2-VCAN (**F**), Mono3-FCGR3A (**G**), DC1-CD1C (**H**), DC2-CLEC4C (**I**), and Neu-FCGR3B (**J**). **K**, Heatmap showing the gene set variation analysis (GSVA) scores for hallmark pathway gene sets enriched in myeloid cell clusters. Red: upregulation; blue: downregulation. **L**, Proportions of myeloid cell clusters in pPBMC and hPBMC. DC: dendritic cells.

After performing unsupervised re-clustering, we discerned a total of seven distinct clusters of myeloid cells (Figure 5A-D). Utilizing established gene signatures of myeloids, we identified four monocyte clusters, two DC clusters, and one neutrophil cluster. Mono1-CD14, Mono2-VCAN, and Mono4-THBS1 exhibited high expression levels of classical monocyte marker genes (*CD14*, *S100A9*, *S100A8*), representing the classical monocyte. Mono3-FCGR3A was characterized by elevated expression of non-classical monocyte marker genes (*FCGR3A*, *CDKN1C*), defining the non-classical monocyte. DC1-CD1C demonstrated strong expression of *CLEC9A* and *CD1C*, annotated as cDC2. DC2-CLEC4C featured high expression levels of CLEC4C and IL3RA, annotated as plasmacytoid DC. Neu-FCGR3B notably expressed *FCGR3B* and *CXCL8*, as well as myeloid-derived suppressor cell (MDSC) feature marker genes (*IL4R*, *CD274*), representing the MDSC-like neutrophil phenotype (Figure 5E).

We then conducted GO analysis to delineate the functions of myeloid cells in PBMCs. Mono2-VCAN exhibited enrichment in genes associated with “phagocytosis, leukocyte cell-cell adhesion, cell chemotaxis, leukocyte migration, and response to molecule of bacterial origin”, illustrating its roles in classical monocyte functions like phagocytosis, antibacterial activity, migration, and chemotaxis (Figure 5F). Mono3-FCGR3A harbored genes related to “regulation of lymphocyte activation, regulation of cell-cell adhesion, regulation of immune effector process”, indicative of an immunomodulatory role (Figure 5G). DC1-CD1C showed gene enrichment in “antigen processing and presentation, cellular response to interferon-gamma, antigen receptor-mediated signaling pathway”, suggesting its involvement in antigen presentation and response to IFN-γ (Figure 5H). DC2-CLEC4C demonstrated enrichment in “cellular response to interferon-gamma, regulation of immune effector process, interferon-gamma-mediated signaling pathway, regulation of inflammatory response”, highlighting its function in immunomodulation and IFN-γ response (Figure 5I). Neu-FCGR3B was associated with genes in “neutrophil degranulation, neutrophil mediated immunity, phagocytosis, negative regulation of immune system process, cellular response to interferon-gamma”, suggesting a potential role in immune inhibition and IFN-γ response (Figure 5J). Additionally, we performed GSVA of hallmark pathways. Mono4-THBS1 showed gene enrichment in “hypoxia, angiogenesis”, while Neu-FCGR3B exhibited genes related to the “interferon gamma response, interferon alpha response” (Figure 5K).

Finally, we assessed the differences in myeloid subpopulations between patients and the healthy donor. It was observed that the cell proportions of Mono4-THBS1 and Neu-FCGR3 were higher in patients (Figure 5L). In summary, our findings demonstrated that myeloid subpopulations possess distinct functions within PBMCs.

## Discussion

In this study, we comprehensively profiled the immune cell landscape in PBMCs from bladder cancer patients and a healthy donor using scRNA-seq analyses. Our investigations revealed a distinct composition of immune cell types between the two groups, highlighting notable differences between patients and the healthy donor.

Among T cell clusters, we identified two clusters of naive T cells, one cluster of help T cells, one cluster of Tregs, four clusters of cytotoxic T cells, and two clusters of Texs. Notably, CD8-T2-GZMK from patients exhibited significantly higher expression levels of *LAG3*, *HAVCR2*, and *CTLA4* compared to those from the healthy donor. Lymphocyte activation gene-3 (LAG-3), an inhibitory receptor expressed on activated T cells, plays a crucial immunosuppressive role by inhibiting T cell proliferation and activation (13). In non-small-cell lung adenocarcinomas, elevated levels of LAG-3 have been linked with poorer overall survival (14). Similarly, infiltration of LAG-3^+^ cells in MIBC correlates with CD8^+^ T cell dysfunction, adverse outcomes, and chemotherapeutic resistance (15). Given the significant role of LAG-3 in T cell exhaustion, various LAG-3 modulating agents are currently undergoing clinical trials. T cell immunoglobulin and mucin-domain containing-3 (TIM-3), encoded by *HAVCR2* and expressed on diverse immune cells including T cells, NK cells, DCs, and macrophages, is another critical inhibitory receptor (16). High expression levels of TIM-3 are negatively associated with patient survival in multiple solid tumors, such as bladder cancer, lung cancer, gastric cancer, ovarian cancer, cervical cancer, hepatocellular carcinoma, and clear cell renal cell carcinoma (17). Consequently, TIM-3 blockade has emerged as a promising target due to its extensive immune-suppressive effects (16). Since the FDA approval of atezolizumab for platinum-refractory, advanced bladder cancer in 2016, several PD-1/PD-L1 inhibitors have been authorized for treating bladder cancer patients (2). However, only a limited number of patients have benefited from these ICIs (18). Our findings suggested that LAG-3 and TIM-3 could be new potential targets for ICIs in bladder cancer therapy. Another intriguing subset of Texs is CD8-T7-STMN1, characterized by high expression of *ITGAE* (*CD103*), *CD38*, and *STMN1*. Integrin αE (CD103), also known as CD103, is a recognized marker for tissue-resident T cells (19). CD8^+^CD103^+^ tissue-resident memory T cells have been identified as a crucial T cell subset associated with poorer clinical outcomes in cutaneous squamous cell carcinoma (20). Stathmin-1 (STMN1) is known for its role in promoting cell proliferation and differentiation through its involvement in the assembly of microtubules and spindles (21). This suggests that CD8-T7-STMN1 may represent active tumor-infiltrating lymphocytes circulating in the peripheral blood. Further investigation of CD8-T7-STMN1 in PBMCs, such as TCR repertoire profiling, could shed light on the properties of intratumoral T cells, thereby enhancing the potential of “liquid biopsies”.

NK cells are pivotal in the innate immune response against tumors. Historically, two primary subsets of NK cells in peripheral blood have been distinguished by their differential expression of CD56 and CD16, termed CD56^bright^ and CD56^dim^ NK cells (22,23). CD56^bright^ NK cells are characterized by high expression of the inhibitory MHC class I-binding receptor CD94/NKG2A and low levels of perforin and granzymes A and B (24). These cells are known for producing a plethora of immunomodulatory cytokines and exhibiting relatively low natural cytotoxicity (23,25). Conversely, CD56^dim^ NK cells demonstrate high expression of perforin and granzymes, but low levels of CD94/NKG2A (23). In comparison to their CD56^bright^ counterparts, CD56^dim^ NK cells play a more potent role in cytotoxic immune responses (26). Our study uncovered additional NK cell subsets beyond these two major groups in peripheral blood. NK1-SPON2, with its moderate expression level of *NCAM1*, might represent an intermediate state between CD56^bright^ and CD56^dim^ NK cells, potentially serving dual roles in immunomodulation and cytotoxicity, as suggested by GO analysis. Notably, NK3-CMC1, which was found to be highly expressed in bladder cancer patients, showed elevated levels of *TIGIT*. TIGIT receptor interactions with its ligands can suppress the cytolytic activity of NK cells, leading to their exhaustion (27,28). The blockade of TIGIT has been shown to prevent NK cell exhaustion and enhance their anti-tumor immunity (27). Furthermore, our data indicated that active receptor and granzyme genes were expressed at lower levels in bladder cancer compared to the healthy donor, suggesting that the phenotypes of NK3-CMC1 might be altered by tumor progression.

B cells represent a significant lymphocyte population in PBMCs. Within the B cell clusters, Bcell2-TCL1A and Bcell3-MS4A1 exhibited high expression of inhibitory receptor marker genes. Bcell2-TCL1A was distinguished by elevated expression levels of *LAIR1* and *BTLA*. Leukocyte-associated immunoglobulin-like receptor-1 (LAIR-1), a type I transmembrane glycoprotein prevalent on naive B cells, is implicated in inhibiting B cell proliferation and activation (29). The B and T lymphocyte attenuator (BTLA), a CD28 superfamily member, predominantly expressed on B and T cells (30), negatively regulates B cell functions such as cytokine secretion and co-stimulatory molecule upregulation by binding with herpesvirus entry mediator (HVEM) (30,31). GO analysis suggested that Bcell2-TCL1A may possess immune regulatory capabilities. Bcell3-MS4A1 showed heightened expression of genes encoding FcγRIIB, FCRL, Siglec-10, LILRB, and CD72. These molecules, which contain immunoreceptor tyrosine-based inhibition motifs (ITIMs), are known to downregulate B cell antigen receptor (BCR) signaling (32). Accordingly, GO analysis indicated that Bcell3-MS4A1 might play a suppressive role in immune regulation. Notably, the cell proportions of Bcell2-TCL1A and Bcell3-MS4A1 were found to be increased in patients compared to the healthy donor, suggesting that further investigation into these subsets could elucidate the impact of tumors on B cell behavior.

Monocytes, integral to the innate immune response, continuously circulate in the bloodstream and transition to various tissue types. Their dynamic roles in immune regulation are critical in tumor development. Their first role involves enhancing NK cell recruitment and activation and clearing tumor materials from the vasculature (33). As key sources of tumor-infiltrating immunocytes, like tumor-associated macrophages and DCs, monocytes are instrumental in promoting tumor immune escape by evolving into immune regulatory cells (34). Additionally, their chemokine-driven attraction to tumor metastases promotes the proliferation of tumor cells (35). This multifaceted nature of monocytes underscores their subset heterogeneity. Our analysis revealed that Mono1-CD14, Mono2-VCAN, and Mono4-THBS1 represented classic monocyte phenotypes. Among these, the proportion of Mono4-THBS1 was notably increased in patients, with GSVA implicating Mono4-THBS1 in the promotion of tumor hypoxia and angiogenesis. In colorectal cancer, tumor-enriched FCN1^+^ monocyte-like cells exhibited a high resemblance to blood CD14^+^ monocytes, likely representing a monocyte population that migrates into tumors and adopts a tumor-specific transcriptional program (36). This suggests that Mono4-THBS1 might be a source of these tumor-enriched monocyte-like cells.

Neutrophils exert a range of effects on tumor progression and metastasis, and the neutrophil-to-lymphocyte ratio (NLR) is notably significant for prognostic evaluation in urological tumors (37). Our findings indicated that Neu-FCGR3B represented low-density neutrophils (LDNs), and there was an increased cell proportion of Neu-FCGR3 in patients. Initially identified in the peripheral blood of patients with autoimmune diseases in 1986, LDNs have subsequently been confirmed to proliferate in the peripheral blood of tumor patients (38). Neu-FCGR3B exhibited high expression levels of *IL4R*, *CD274*, suggesting a potential inhibitory role in immune regulation. PD-L1^+^ neutrophils are capable of suppressing the proliferation and activation of T cells (39). This implies that Neu-FCGR3B might negatively influence the anti-tumor response and could serve as a potential predictive marker for bladder cancer.

## Conclusion

In summary, our study paints a comprehensive portrait of the immune cell landscape within PBMCs from bladder cancer patients and a healthy donor, highlighting both shared and unique transcriptional characteristics. However, due to the high cost and technical limitations of scRNA-seq, our sample size was limited and the sensitivity of gene detection was constrained. Consequently, larger cohort studies are essential to replicate our research and corroborate our findings. Collectively, our data provided a valuable resource for future investigations into therapeutic targets and biomarkers for bladder cancer immunotherapy.

## Supplementary Material

Click to view [pdf].
