# Supplementary material for: Single-cell analysis reveals potential therapeutic markers of peripheral blood mononuclear cells from bladder cancer patients
Source: Braz J Med Biol Res. 2025 May 9;58:e14002. doi: 10.1590/1414-431X2025e14002 (PMC12068768; doi:10.1590/1414-431X2025e14002)
Supplement: Supplementary file 1 [file 1414-431X-bjmbr-58-e14002-suppl.pdf]

Figure S1. Cell proportions of major peripheral blood mononuclear cells in patients (pPBMC) and three healthy donors (hPBMC) (for validation). A, Proportions of T cells in the two groups. B, Proportions of natural killer (NK) cells. C, Proportions of B cells. D, Proportions of monocytes. E, Proportions of dendritic cells (DC). F, Proportions of neutrophils. Data are reported as medians and interquartile range; Wilcoxon rank-sum test.

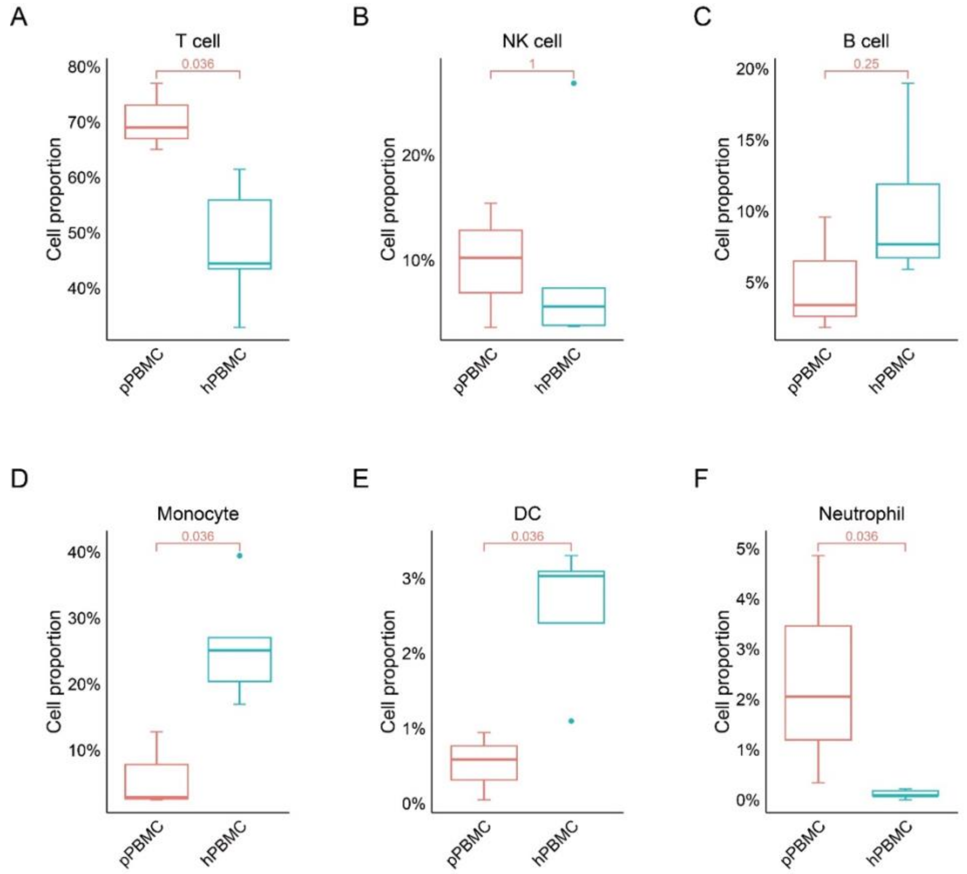

Table S1. Demographic and clinical information of bladder cancer patients.

Patient ID	Gender	Age	Sample	TNM	History of treatment
P01	Male	66	PBMC	T1N0M0	Treatment naive
P02	Male	63	PBMC	T2bN0M0	Treatment naive
P03	Male	64	PBMC	T2aN0M0	Treatment naive
P04	Male	74	PBMC	T3aN0M0	Treatment naive

PBMC: peripheral blood mononuclear cells; T: tumor; N: node; M: metastasis.
